# Destructive impact of t-lymphocytes, NK and mast cells on basal cell layers: implications for tumor invasion

**DOI:** 10.1186/1471-2407-13-258

**Published:** 2013-05-25

**Authors:** Hongyan Yuan, Yi-Hsuan Hsiao, Yiyu Zhang, Jinlian Wang, Chao Yin, Rong Shen, Yiping Su

**Affiliations:** 1 Department of Oncology, the Affiliated Nanjing Maternity and Child Health Care Hospital, Nanjing Medical University, Nanjing, China; 2 Institute of Medicine, Chung Shan Medical University, Taichung, Taiwan; 3 Department of Obstetrics and Gynecology, Changhua Christian Hospital, Changhua, Taiwan; 4 Johns Hopkins Bloomberg School of Public Health, Johns Hopkins University, Baltimore, Maryland, USA; 5 Department of Oncology, Georgetown University, Washington, DC, USA; 6 Georgetown University School of Medicine, Washington, DC, USA

**Keywords:** Tumor, Infiltration, Destruction, Immune cells, Invasion

## Abstract

**Background:**

Our previous studies have suggested that the primary impact of immune cell infiltration into the normal or pre-invasive tissue component is associated with the physical destruction of epithelial capsules, which may promote tumor progression and invasion. Our current study attempted to further verify our previous observations and determine the primary type(s) of infiltrating immune cells and the possible mechanism associated with physical destructions of the epithelial capsules.

**Methods:**

In total, the study was conducted with 250 primary breast and prostate tumors, the primary immune cell of cytotoxic T-lymphocytes (CTL), Natural killer cells (NK) and Mast cells were analyzed by immunohistochemistry, fluorescent labeling and apoptosis assay. qRT-PCR was used for gene expression analysis. Our current study assessed the physical disruption of these immune cells and potential impact on the epithelial capsule of human breast and prostate tumors.

**Results:**

Our study yield several clinically-relevant findings that have not been studied before. (1) A vast majority of these infiltrating immune cells are distributed in the normal-appearing or pre-invasive tissue components rather than in invasive cancer tissues. (2) These cells often form rings or semilunar structures that either surround focally-disrupted basal cell layers or physically attach to the basal cells. (3) Basal cells physically associated with these immune cells generally displayed distinct signs of degeneration, including substantially elevated apoptosis, necrosis, and reduced tumor suppressor p63 expression. In contrast, luminal cells overlying focally disrupted basal cell layers had a substantially increased proliferation rate and elevated expression of stem cell markers compared to their adjacent morphologically similar counterparts that overlie a non-disrupted capsule.

**Conclusion:**

Our findings suggest that at the early stage of tumor invasion, CTL, NK and Mast cells are the main types of tumor infiltrating immune cells involved in focal degenerative products in the tumor capsules. The primary impact of these infiltrating immune cells is that they are associated with focal disruptions of the tumor capsule, which selectively favor tumor stem cells proliferation and invasion.

## Background

The impact and clinical significance of tumor-infiltrating immune cells remain a hot topic after decade of debate. A large number of publications have reported that the direct physical contact between infiltrated immune cells and tumor cells is associated with the physical destruction of associated tumor cells, reduction of the tumor size, and significantly improved clinical prognoses [1-6]. However, an increasing number of publications show that increased infiltration of immune cells may promote tumor progression and invasion. Previous reports that stage and histopathologically matched pre-invasive prostate and esophageal tumors with increased immune cell infiltration have a significantly higher frequency of subsequent progression to invasive lesions, compared to their counterparts without immune cell infiltration [7-9]. It has also been reported: ***(a)*** macrophages can enhance cancer cell migration through secretion of chemotactic and chemokinetic factors that promote fibrillogenesis and angiogenesis, allowing tumor cells to track along collagen fibers and blood vessels [10,11], ***(b)*** macrophages ingest tumor cells induce a mixture of genetic materials and create a hybrid phenotype which can metastasize to remote sites to form new colonies [12], ***(c)*** T-lymphocytes promote invasion and metastasis by regulating tumor-associated macrophages [13]; ***(d)*** infiltration of immune cells can export growth factors and other proliferation-related molecules to associated tumor cells through direct physical contact and promote epithelial-mesenchymal transition (EMT) and cell motility [14,15].

The contradictory observations regarding the impact of tumor-infiltrating immune cells have caused confusions in judging the clinical implications of aberrant infiltration of immune cells within tumor tissues. In addition, as immune cell-mediated lysis or cytotoxic assays are almost exclusively conducted on cell cultures or animal models, these *in vitro* assays cannot fully mimic the intrinsic events in human carcinogenesis [16-18]. It has been well documented that the immune-surveillance systems differ significantly in human and animals [19,20].

We have speculated that these contradictory reports and statements may result from different tumor stages, in which infiltrating immune cells may be associated with differential consequences depending on the cell type. To validate our speculation, our previous studies compared the pattern and frequency of physical association of tumor-infiltrating immune cells with basal and luminal cells of breast and prostate tumors that harbor both pre-invasive and invasive components. Our studies revealed that: (1) over 90% of infiltrating immune cells were distributed in the normal or pre-invasive tissue component, while fewer than 10% were in the invasive tissue component, (2) within the normal or pre-invasive tissue component, over 90% of the epithelial structures with a focally disrupted epithelial capsule were associated with infiltrating immune cells, compared to about 30% in epithelial structures with a non-disrupted capsule, (3) a vast majority of infiltrating immune cells were located on or near the site of focally disrupted epithelial capsules, and (4) epithelial cells overlying focally disrupted capsules often show a substantially increased proliferation rate and often form finger- or tongue-like projections invading the adjacent stroma [21-25]. Based on these and other findings, we have hypothesized that the primary impact of immune cell infiltration into normal or pre-invasive tissue component is the physical destruction of epithelial capsules, which may promote tumor progression and invasion [26,27].

Our current study attempted to further verify our previous observations and determine the primary type(s) of infiltrating immune cells and the possible mechanism(s) associated with physical destructions of the epithelial capsules. As it has been well documented that: (1) CTL, natural killer (NK) and Mast cells are the primary immune cell types for detecting and eliminating degenerated and altered host cells and (2) these three cell types harbor similar cytotoxic granules and share the same mechanism for the exocytosis of their granules [28-31], we have hypothesized they may be preferentially localized on degenerated basal cell layers and function coordinately in the physical destruction of degenerated epithelial capsules.

## Methods

Formalin-fixed, paraffin-embedded human breast tissue (N = 150) were retrieved from the files of the Department of Pathology and Department of Oncology, the Affiliated Nanjing Maternity and Child Health Care Hospital, Nanjing Medical University. Human prostate (N = 100) tumor tissue blocks were obtained from Department of Pathology, Affiliated Jiangsu people’s hospital, Nanjing Medical University with an IRB approval from Nanjing Medical University.

Serial 5-7 μm sections were made from the breast and prostate tumor tissue blocks. The first and last sections from each block were stained with Hematoxylin & Eosin (H&E) for morphological classification using published criteria. Double immunohistochemistry was applied to assess the potential impact of infiltrating immune cells on other cell types and structures using previously published protocol [32]. The secondary antibody, ABC detection kit and diaminobenzidine chromogen kit were obtained from Vector Laboratories (Burlingame, CA, USA). The AP red-chromogen kit was purchased from Zymad Laboratories (South San Francisco, CA, USA). Negative control slides (IgG only) were included in each individual analysis.

Immune cell aggregates and tumor infiltrating immune cells were elucidated with leukocyte common antigen (LCA, clone: 2B11 + PD7/26; Dako, Carpinteria, CA, USA), CD8 (clone: C8144B), CD16 (clone: DJ130c) and Mast cell tryptase (clone: AA1) (Dako). The breast myoepithelial and prostate basal cells were identified with smooth muscle actin (SMA; clone: 1A4; Sigma, USA) and cytokeratin (CK34βE12 (clone: M0630; Dako) respectively. The focal capsule disruptions were defined as the presence of a physical gap in a given myoepithelial or basal cell layer that is larger than the combined size of at least three epithelial cells and in at least three consecutive sections. Physical signs of degeneration-related changes were defined as the loss of expression of phenotypic markers, vacuolation, fragmentation, swelling, nuclear membrane breakage, chromatin condensation, atrophy, and necrosis. To assess the potential impact of physical association between infiltrated immune cells and basal or luminal cells, immunostained sections and photographs were independently reviewed by three investigators. A given cell was considered immunoreactive if distinct immunoreactivity was consistently present in its cytoplasm, membrane or nucleus while absent in all negative controls. All infiltrated immune cell aggregates in each case were counted, the frequencies of these aggregates within pre-invasive and invasive tissue components were statistically compared by the Pearson’s Chi-square test. To compare the frequencies of cytological signs of degeneration in basal and luminal cells that are physically associated with infiltrated immune cells, digital images were taken in 3–5 randomly selected basal cell layers and associated luminal cells subjacent to or surrounded by infiltrated immune-cells. Digital images were enlarged and viewed on a computer to detect potential signs of degeneration defined as above. The total numbers of cells counted and cells with cytological signs of degeneration in each group were added and averaged, the averaged frequencies were statistically compared by the Pearson’s Chi-square test. Statistical significance was defined as *p* < 0.05.

To assess the biological and molecular changes along with potential mechanisms caused by the physical association between infiltrating immune cells and the basal or luminal cells, the following technical approaches were utilized:

(A) **Apoptosis assay.** Tissue sections from 10 cases with a high frequency of degeneration-related changes in the basal cell layers were subjected to apoptosis detection with Apoptosis Detection Kit (CHEMICON International, USA) according to the manufacturer’ protocol. After wash, the sections were staining for CK34βE12 to determine the histological origin of apoptotic cells.

(B) **The expression status of tumor suppressor p63 in the basal cell layers:** p63 expression in basal cells within non-disrupted and focally disrupted residual layers were compared by double immunostaining of CK34βE12 and p63. To verify the sub-cellular localization of basal cell phenotypic makers CK34βE12 and p63, sets of adjacent human prostate tissue sections from 10 selected cases were double immunostained for CK34βE12 and p63 (clone: 4A4; Cell Marque, Rocklin, CA, USA). The antigen-antibody complexes were distinguished by different secondary antibodies labeled with different fluorophores (DyLight 488 and Dylight 549; KPL, Gaithersburg, MD, USA) according to the manufacturer’s instructions. Immunostained sections were examined and digital images were taken in a fluorescent microscope (Fluoview 300; Olympus America, Inc., Center Valley, PA, USA).

(C) **Proliferation index and lymphatic duct density:** To further assess the potential impact of infiltrating immune cells on cell proliferation and angiogenesis, tissue sections from 30-selected cases were double immunostained for CK34βE12 and a cell proliferation marker, Ki-67 (clone: MM1; Dako). The proliferation status in epithelial cells surrounded by infiltrating immune cells were compared to those cells that were distant from infiltrating immune cells. In addition, special attention was paid to identify whether proliferating cells were preferentially located on the site of focally disrupted basal cell layers.

(D) **Microdisection and Quantitative Real-Time PCR (qRT-PCR):** Five unstained adjacent sections or immunostained sections with pre-invasive tumors showing extensive focal capsule disruptions and immune cell infiltration were deparaffinized and lightly stained with hematoxylin. Guided by immunostained sections, morphologically similar pre-invasive lesions with and without extensive focal capsule disruptions and immune cell infiltration were microdissected and subjected to RNA extraction by RNeasy FFPE Kit (Qiagen) according to the manufacturer’s instructions. Total RNA (0.5 μg) was reverse transcribed in a total volume of 20 μl using the Omniscript RT kit (Qiagen). Quantitative PCR was performed in triplicate in an ABI-Prism 7700 instrument (Applied Biosystems, Foster City, CA,USA) using QuantiTect Sybr green (Qiagen, Gaithersberg, MD, USA) according to the manufacturer’s protocol. The appropriate primer sets were listed in Additional file 1: Table S1. Amplification was confirmed by ethidium bromide staining of the PCR products on a 2% agarose gel. The expression of each target gene was based on five pooled samples, and normalized by GAPDH RNA expression, expressed as 2^-ΔCt^, where Ct is the threshold cycle and ΔCt = Ct^Target^ - Ct ^GAPDH^.

### Statistical analysis

Student’s test was performed for two group comparisons. The data presented represent mean ± SEM. P-value of less than 0.05 was considered to be statistically significant.

## Results

The sub-cellular localization and cell types that reacted to the antibodies were consistent with those of published data and specifications of manufactures. All negative controls were consistently devoid of distinct immunoreactivity. The results were highly consistent in duplicates or triplicates. The basal and luminal cells were distinguishable by morphology and by immunohistochemistry in all study cases. The basal cell population is characterized by its spindle or elongated shape with densely stained nucleus, CK34βE12 staining in the cytoplasm, and p63 staining in nucleus. The luminal cell population is characterized by its round or oval shape with lightly stained nucleus harboring distinct nucleoli. It is devoid of CK34βE12 and p63 expression. CD16, CD56, Fox3p, Mast, and neutrophils cells are primarily known as lymphocytes; thus, they have been collectively referred as infiltrated lymphocytes below. Both the prostate basal and breast myoepithelial cells are referred to as basal cells below.

**Figure 1:**
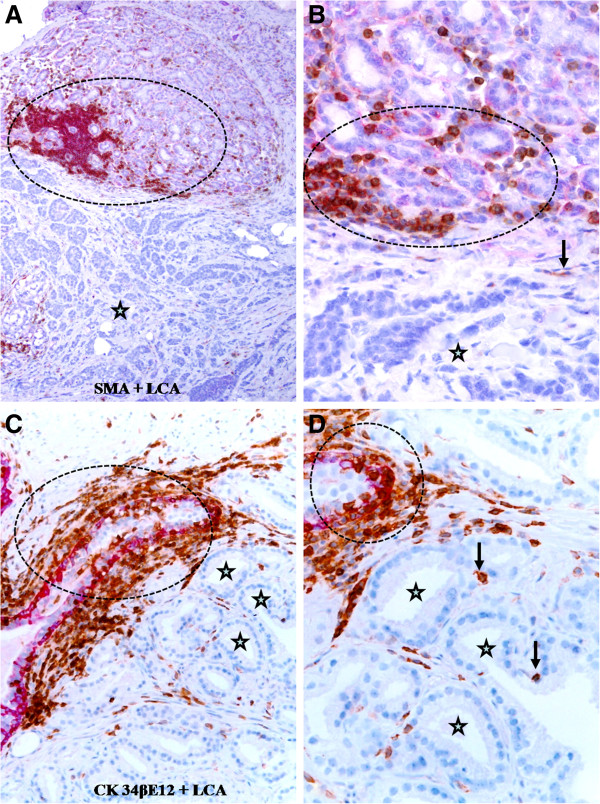
**Lymphocyte aggregates in normal or hyperplastic breast and prostate tissues.** Paraffin-embedded human breast **(A-B)** and prostate **(C-D)** tissue sections were double immunostained for smooth muscle actin (SMA; a myoepithelial marker; red) plus leukocyte common antigen (LCA; brown) or cytokeratin (CK) 34βE12 (a basal cell marker; red) plus LCA. Note that lymphocyte aggregates (circles) are exclusively associated with normal or hyperplastic epithelial structures, whereas the invasive component (stars) only harbors a few isolated lymphocytes (arrows). **A** and **C**: 100X. **B** and **D**: a higher (300X) magnification of **A** and **C**, respectively.

Similar to our previous studies, lymphocyte aggregates were almost exclusively in some normal appearing or pre-invasive breast and prostate epithelial structures, in which many infiltrated lymphocytes were physically attached to the basal cell layers that were often focally disrupted or substantially attenuated (Figure 1). In sharp contrast, significantly fewer infiltrated lymphocytes were seen within the invasive tissue component, and over 95% of the invasive cancer cells had no direct physical contact with infiltrated lymphocytes. In a total of 154 lymphocyte aggregates detected near epithelial structures, 147 (95.5%) were associated with normal or pre-invasive epithelial structures with focally disrupted capsules, only 7 (4.5%) were located at the invasive cancer component (Table 1; *p* < 0.01).

**Table 1:** Distribution of lymphocyte aggregates

**Number of aggregates**	**Invasive cancer component**	**Focally disrupted capsules**	**p-value**
154	7 (4.5%)	147 (95.5%)	<0.01

**Figure 2:**
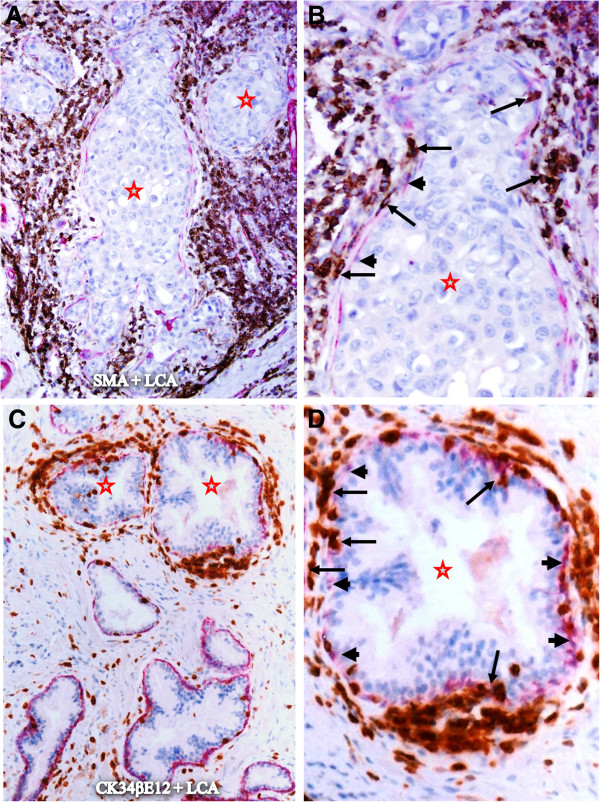
**Lymphocyte infiltration within in situ breast and prostate cancer tissues.** Paraffin-embedded human breast **(A-B)** and prostate **(C-D)** tissue sections were double immunostained for SMA (red) plus LCA (brown) or CK34βE12 (a basal cell marker; red) plus LCA. Note that although these in situ breast and prostate tumor nests (stars) are almost completely surrounded by infiltrating immune cells (arrows), a vast majority of the tumor cells are physically separated from the surrounding infiltrating immune cells by the tumor capsule (the myoepithelial or basal cell layer) (arrowheads). **A** and **C**: 100X **B** and **D**: a higher (300X) magnification of **A** and **C**, respectively.

Within the *in situ* breast and prostate cancer tissue component, infiltrating immune cells often formed ring or semilunar structures, completely or partially surrounding the epithelial tissue. As shown in Figure 2, many infiltrating immune cells are physically attached to the epithelial capsules, while the epithelial component is generally free of infiltrating immune cells if the surrounding epithelial capsule is not focally disrupted.

**Figure 3:**
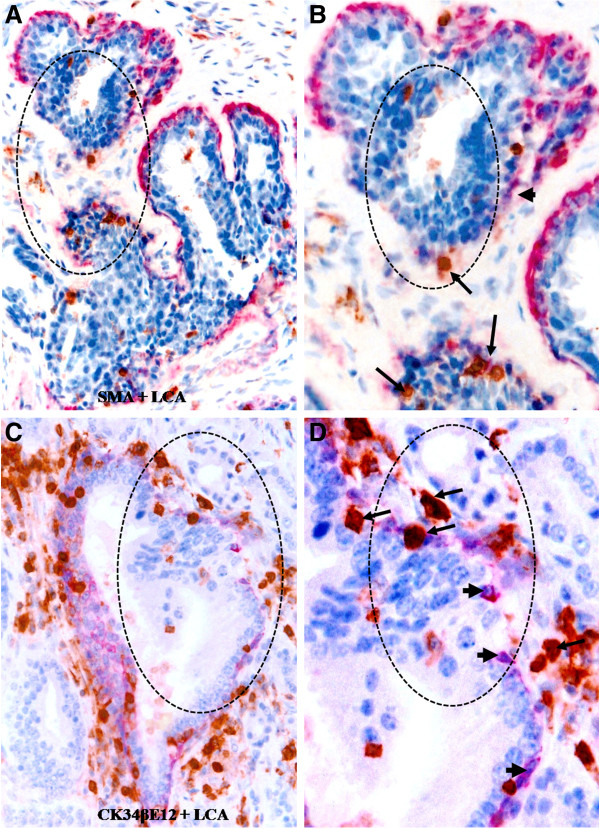
**Lymphocyte infiltration at sites of focal capsule disruptions of normal appearing tissues.** Paraffin-embedded human breast **(A-B)** and prostate **(C-D)** tissue sections were double immunostained for SMA (red) plus LCA (brown) or CK34βE12 plus LCA. Note that most infiltrating lymphocytes (arrows) are located on the site of focally disrupted epithelial capsules (circles), in which the overlying epithelial cells formed tongue-like projections invading the adjacent stroma. **A** and **C**: 100X. **B** and **D**: a higher (400X) magnification of **A** and **C**, respectively.

In normal or hyperplastic epithelial structures with a focally disrupted epithelial capsule, a majority of infiltrating immune cells was located on or near the site of focal capsule disruptions. This observation is shown in Figure 3, in which the overlying epithelial cells formed finger- or tongue-like projections physically associated with the adjacent stromal tissue.

**Figure 4:**
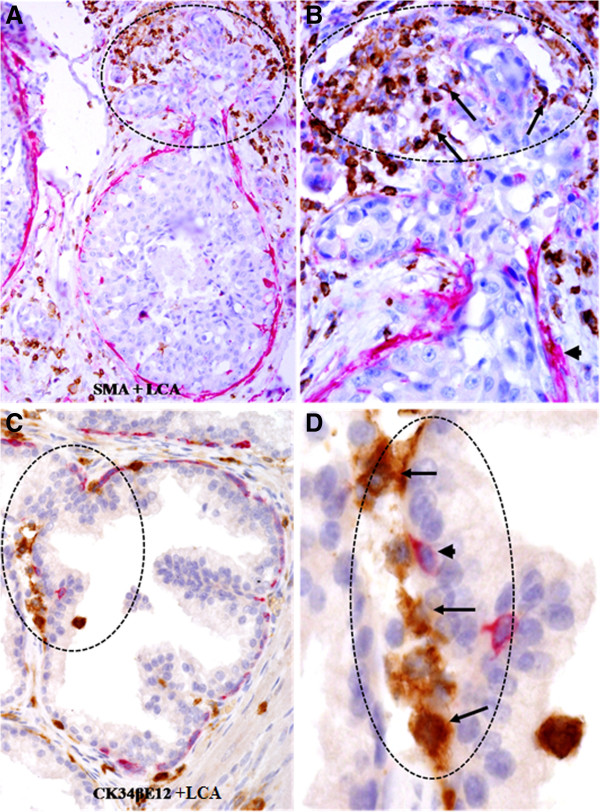
**Lymphocyte infiltration at sites of focal capsule disruptions of in situ cancer tissues.** Paraffin-embedded human breast **(A-B)** and prostate **(C-D)** tissue sections were double immunostained for SMA (red) plus LCA (brown) or CK34βE12 plus LCA. Note that most infiltrating lymphocytes (arrows) are located on the site of focally disrupted epithelial capsules (circles), in which the overlying tumor cells formed tongue-like structures invading the adjacent stroma. **A** and **C**: 100X. **B** and **D**: a higher (400X) magnification of **A** and **C**, respectively.

In pre-invasive cancerous epithelial structures with a focally disrupted tumor capsule, infiltrating immune cells were also preferentially located on the site of focally disrupted capsules. As shown in Figure 4, the tumor capsules display distinct signs of degenerative changes, including attenuation, fragmentations and disruptions; whereas the overlying epithelial cells appear to have an elevated proliferation rate, judged from the increased cell density.

**Figure 5:**
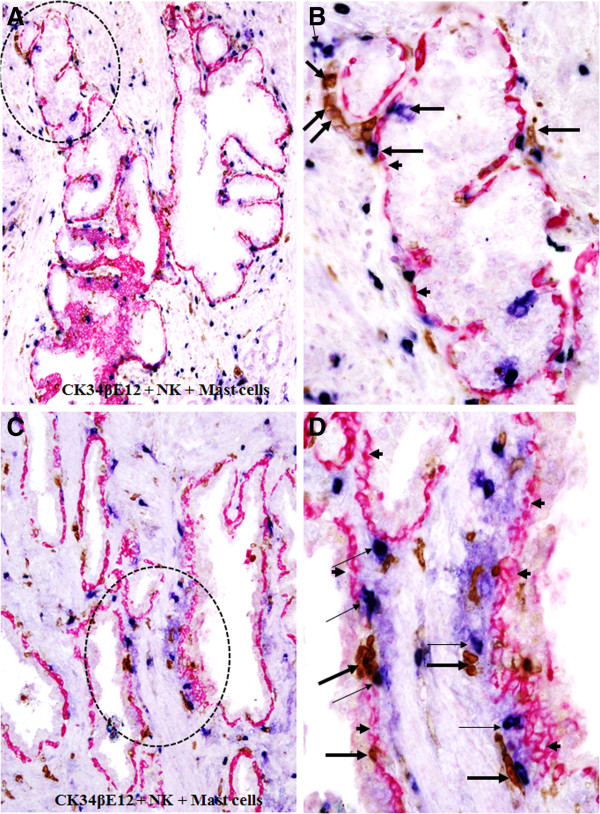
**Mast and NK cell infiltration in normal or hyperplastic prostate tissues.** Paraffin-embedded human prostate tissue sections were triple immunostained with markers for CK34βE12 (red), NK (brown), and Mast (blue) cells. Note that a majority of NK (thick arrows) and Mast (thin arrows) are located along the tumor capsule (arrowheads). **A** and **C**: 100X. **B** and **D**: a higher (300X) magnification of **A** and **C**, respectively.

The predominant type of infiltrating immune cells associated with focal capsule disruptions appears to be CTL, which can be easily recognized by their morphology and immunoreactivities to the anti-CD8 antibody. As shown in Figure 5, similar to CTL, a majority of infiltrating NK and Mast cells in the field are physically attached to the epithelial capsules of normal or hyperplastic epithelial structures. At NK and Mast cell attaching site, the associated capsules were generally attenuated, fragmented or disrupted. It was interesting to note that NK and Mast cells often appeared as pairs and localized on the same site.

**Figure 6:**
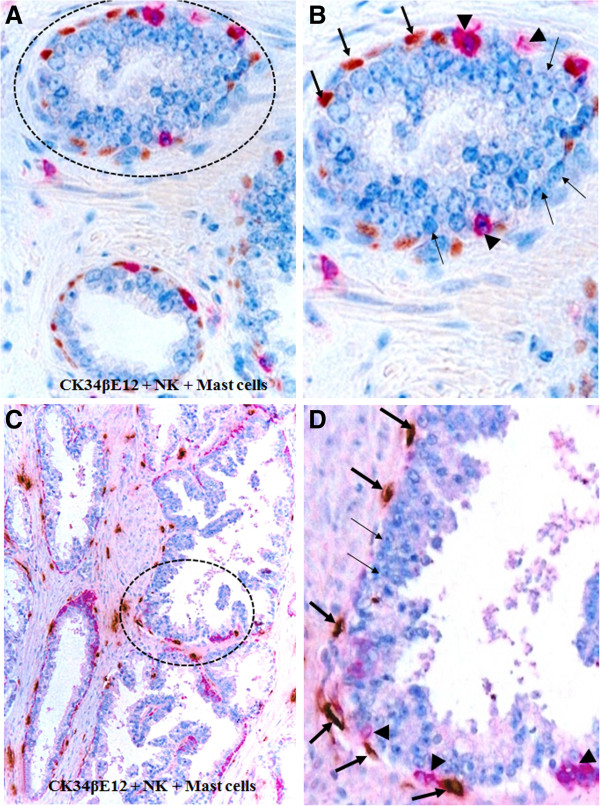
**Mast and NK cell infiltration within in situ prostate tissues.** Paraffin-embedded human prostate tissue sections were triple immunostained with markers for CK34βE12 (red), NK (brown), and Mast (blue) cells. Note again that a majority of NK (thick arrows) and Mast (thin arrows) are located along the tumor capsule (arrowheads).

Similar NK and Mast cell infiltration was also seen in pre-invasive cancerous epithelial structures. As shown in Figure 6, a number of NK and Mast cells lied along with the capsule and physically associated with epithelial cells overlying focally disrupted capsules, whereas the epithelial component is largely free of Mast cells.

**Figure 7:**
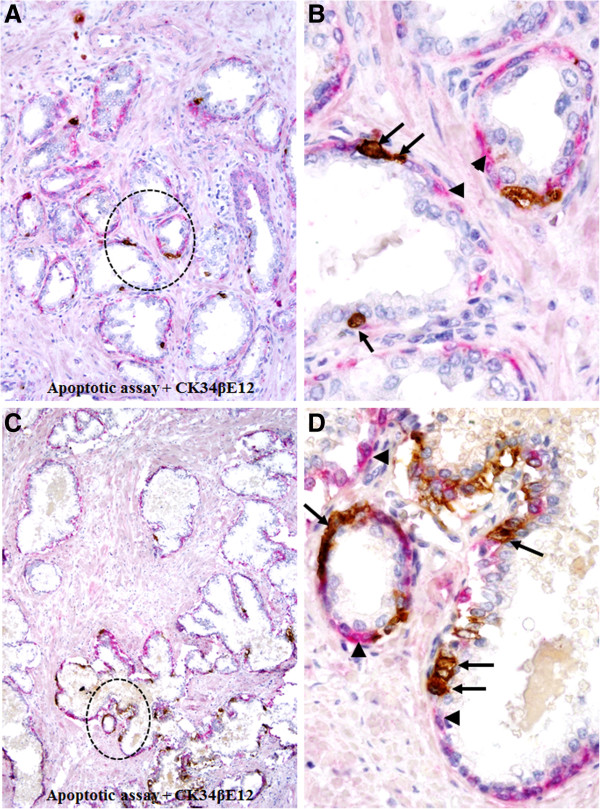
**Elevated apoptosis in focally disrupted basal cell layers.** Paraffin-embedded human prostate tissue sections were subjected to apoptosis assay and also immuostaining for basal cell markers. Note that nearly all apoptotic cells (arrows) are the epithelial capsule element (arrowheads), the basal cells. **A** and **C**: 100X. **B** and **D**: a higher (300X) magnification of **A** and **C**, respectively.

Focal capsule disruptions appear to result from focal degenerative alterations in the constitutional elements of the epithelial capsule. As shown in Figure 7, apoptotic cells are exclusively seen in the basal cell layers surrounding the epithelial component, which is largely devoid of or have significantly fewer apoptotic cells.

**Figure 8:**
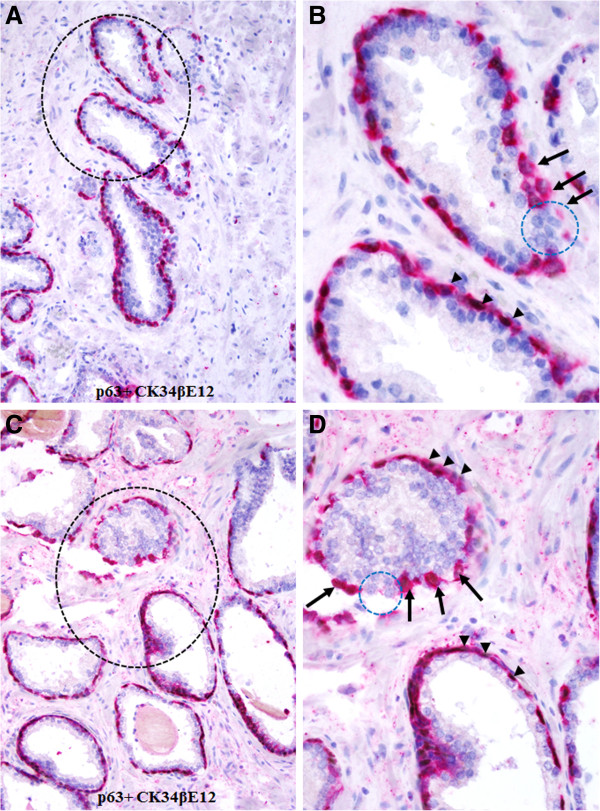
**Loss of p63 expression in basal cells detected by chromogrn-based immunostaing.** Human prostate tissue sections were double immunostained for CK 34βE12 plus p63. Note that a majority of basal cells in non-disrupted layers have the expression of CK 34βE12 plus p63 (arrowheads), whereas nearly all those cells near the focally disrupted (circles) layers are devoid expression of tumor suppressor p63 (arrows). **A** and **C**: 200X. **B** and **D**. A higher (500X) of **A** and **C**, respectively.

**Figure 9:**
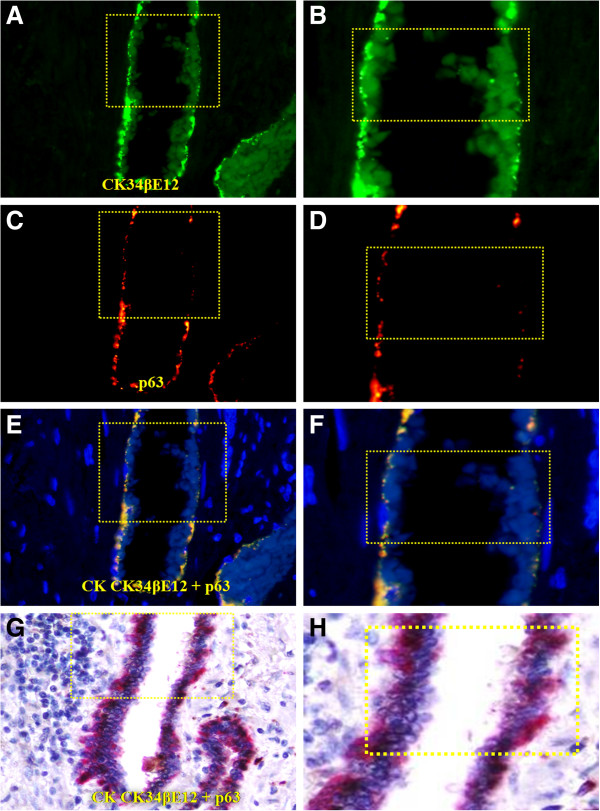
**Loss of p63 expression in basal cells detected by fluorescence-based immunostaining.** A set of three adjacent human prostate tissue sections were double immunostained for CK34βE12 and p63, the antigen-antibody complexes were distinguished with secondary antibodies labeled with different fluorophores **(A-F)** or a red chromogen **(G-H)**. Note that the basal cells near the up-middle have substantially reduced expression of both CK34βE12 and p63. **A** and **C**: 150X. **B** and **D**. A higher (400X) of **A** and **C**, respectively.

Compared to their non-disrupted counterparts, the residual basal cells in focally disrupted capsule have significantly lower expression of tumor suppressor p63. As shown in Figure 8, a majority of the basal cells within non-disrupted capsules express both CK34βE12 and p63, whereas only CK34βE12 is expressed in majority when the capsule is undisrupted. In addition, these undisrupted cells generally display distinct signs of degeneration including the loss of morphological integrity, fragmentations, and necrosis (Figure 8). These degenerative alterations were more clearly appreciable in fluorescent-based immunohistochemistry. As shown in Figure 9, a substantial reduction of the CK34βE12 and p63 expression is seen in a large fragment of a basal cell, suggesting extensive degeneration in this region, which was associated with increased immune cell infiltration (data not shown).

**Figure 10:**
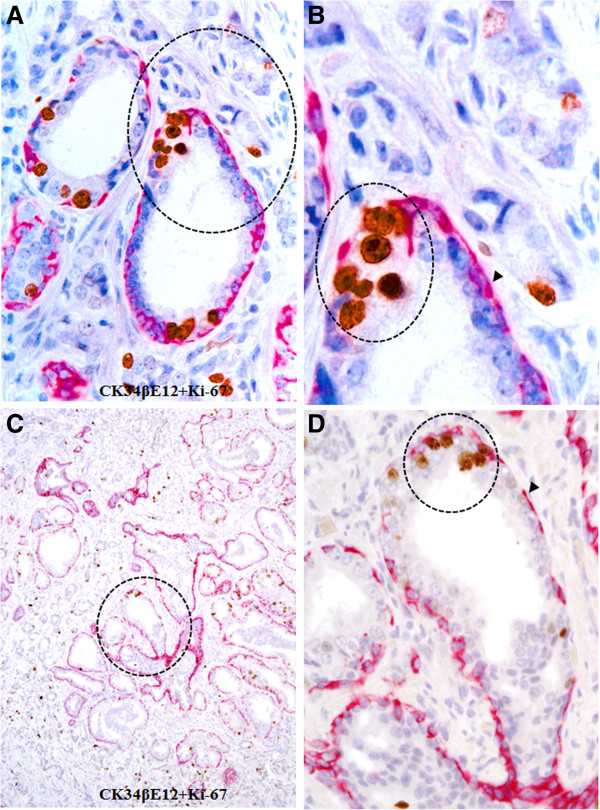
**Elevated proliferation in epithelial cell clusters surrounded by lymphocytes.** Human prostate tissue sections were double immunostained for CK34βE12 plus Ki-67. Note that clusters of multiple proliferating cells are exclusively seen near the site of focally disrupted epithelial capsules (circles). **A** and **C**: 100X. **B** and **D**: a higher (300X) magnification of **A** and **C**, respectively.

In contrast to these degenerative changes the epithelial cells overlying focally disrupted capsules often showed substantially increased cell proliferation. As shown in Figure 10, clusters of multiple Ki-67 expressing cells are exclusively seen at the site of focal capsule disruptions.

**Figure 11:**
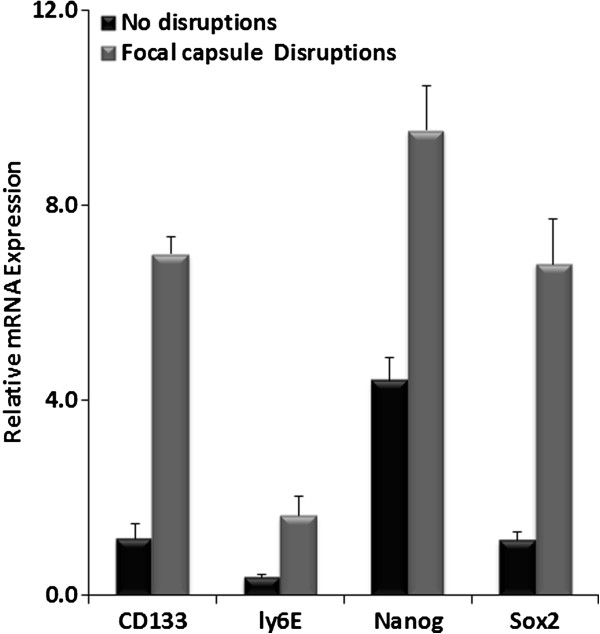
**CD133, ly6E, Nanog and Sox2 mRNA level was determined by qRT-PCR.** All 4 stem marker were elevated in focally disrupted capsules.

Our qRT-PCR results revealed that microdissected luminal cells overlying focal capsule disruptions have significantly higher expression levels of four stem cell markers (CD133, ly6E, Nanog, Sox 2), which were 6-, 4-, 2- and 6-fold higher, respectively, than their undisrupted counterparts at a distance from the site of focal capsule disruptions (Figure 11).

## Discussion

The preferential association of tumor-infiltrating lymphocytes with the basal cell population appears to result primarily from focal degeneration and disruption of the basal cell layers. Our previous studies have consistently shown that compared to its morphologically clear-cut and undisrupted counterpart, a focally disrupted basal cell layer has significantly lower expressions of tumor suppressor p63 and proliferating cell nuclear antigen, while it has significantly higher frequencies of apoptosis and degeneration [33]. As shown in Figures 8 and 9, a vast majority of the basal cells within undisrupted layers express both CK34βE12 and p63, while most basal cells within disrupted layers lack p63 expression with distinct signs of degeneration. Thus, it is very likely that the basal cells in these patients may belong to an “aged” or degenerated population, and that the degradation products of these basal cells may function as self-epitopes to stimulate the production of auto-antibodies or to attract the migration and infiltration of immune cells. Consistent with our speculation, our recent study has revealed that protease-degraded collagen I fragments of the breast tumor capsule function as a specific mediator to attract macrophage and other immune cell infiltration [34].

As the breast and prostate epithelium is normally devoid of both blood vessels and lymphatic ducts and the my epithelial or basal cell layer is the sole source of several tumor suppressors [35-37], a focal degeneration or disruption in a given basal cell layer could cause direct exposure of tumor cells to the tumor microenvirment complexity, and lead to several focal alterations with significant consequences, including: (***a***) local loss of tumor suppressors and paracrine inhibitory functions, which confer epithelial cell growth advantages allowing them to escape from programmed cell death [38]; **(*b***) local increase of immune cells infiltration, could increase of permeability to oxygen, nutrients, and growth factors, which selectively favors the proliferation of progenitor/stem cells [39,40]; (***c***) direct epithelial stromal cell contact, which augments the expression of stromal matrix metalloproteinase (MMP) or other cell adhesion molecules , which facilitate epithelial to mesenchymal transition (EMT) and cell motility [41]; (***d***) direct exposure of the epithelial cells to different cytokines, which facilitates vasculogenic mimicry and tumor angiogenesis [42], and (***e***) direct physical contact between newly formed cell clusters and stromal cells, which stimulates the production of tenascin and other host factors such as hepatocyte growth factor(HGF) that facilitate stromal tissue remodeling and drug resisiance [43-45]. These alterations can individually or synchronously increase proliferation and motility in cells overlying such disruptions. Thus, tumor cell clusters overlying focally disrupted capsules are very likely to represent a population of tumor progenitors with a greater propensity to progress to invasive or metastatic cancer. CD133 and ly6E have been reported as tumor-initiating cells (TIC) markers for breast cancer and pancreatic Cancer [46,47], Nanog and Sox2 are ES cell markers that have been reported in poorly differentiated breast cancer, and correlated with poor survival [48]. Elevated expression of stem cell markers in pre-invasive tumors with extensive focal capsule disruptions and immune cell infiltration suggests that focal capsule disruptions and immune cell infiltration may stimulate or facilitate the exit of stem cells from quiescence, allowing the stem cells to undergo proliferation and invasion into the adjacent stroma or vascular structures.

Our recent studies have consistently revealed that 15-30% of the patients with non-invasive breast or prostate tumors harbor epithelial structures that are morphologically normal or hyperplastic in H&E stained sections, but they show frequent focal capsule disruptions with budding cells in immunohistochemically stained sections [26-31]. The intrinsic entity of these structures is unknown, but they most likely represent a previously undefined malignant phenotype, or the direct precursor of malignant lesions. Our results are consistent with several lines of evidence: ***(1)*** mammary ductal intraepithelial neoplasia (DIN)-flat type is a subtle epithelial alteration characterized by one or a few layer(s) of atypical cells replacing the native epithelium, but loss of heterozygosity (LOH) was detected in 17 of 22 (77%) lesions, and monoclonality was established in the 2 cases analyzed [49]; ***(2)*** LOH was detected in morphologically normal lobules adjacent to breast cancers. In 10 cases with LOH at chromosome 3p22-25 in the carcinoma, 6 displayed the same LOH in adjacent normal lobules [50], and ***(3)*** the prostate tissues in a subset of aged men or normal-appearing prostate tissues adjacent to prostate cancer harbored a DNA phenotype that is identical to invasive and metastasized prostate cancer [51-53]. Together, these findings suggest that those morphologically normal or hyperplastic appearing breast and prostate epithelial structures may have accumulated significant genetic abnormalities, which suggests an early stage of malignant transformation or an increased risk for invasive or metastatic lesions.

The infiltrated host immune cell classification in combine with some other marker may have a prognostic value for tumor invasion and metastasis [54]. As the disruption of the tumor capsule is an absolute prerequisite for tumor invasion and metastasis, our hypothesis, if confirmed, is likely to have significant scientific implications and clinical applications. Furthermore, the elucidation of the molecular profiles of the cell population overlying focal capsule disruptions may lead to the identification of clinically relevant surrogate biomarkers that can be used to distinguish between clinically aggressive and indolent pre-invasive tumors.

## Conclusions

Our current study in breast and prostate tumors has revealed: ***(1)*** 154 total lymphocyte aggregates detected were preferentially associated with pre-invasive structures that have focally disrupted capsules; ***(2)*** infiltrated lymphocytes were predominantly seen at the site of focally disrupted tumor capsules; ***(3)*** a vast majority of the basal cells were immediately subjacent to or physically associated with infiltrating lymphocytes and displayed a wide variety of cytological signs of degeneration; and ***(4)*** focal tumor capsule disruptions coupled with lymphocyte infiltration with the stem cell signature appear to facilitate proliferation and dissociation of the overlying luminal cells. Together, our findings are consistent with our hypothesis: tumor-infiltrating lymphocytes are exclusively or preferentially associated with degenerated basal cells in the initial or early stage of tumor invasion. To our knowledge, this is the first report, which suggests that the primary impact of tumor infiltrating CTL, NK and Mast cells may be associated with the physical disruptions of the tumor capsule. In addition, our findings could offer a reasonable explanation for the contradictory reports and statements regarding the impact and clinical significance of immune cell infiltration into tumor tissues.

## Competing interests

We declare that we have no competing interests.

## Authors’ contributions

Y, H, S and S proposed the hypothesis. Y and H designed and performed the experiments. W, S and S analyzed and interpreted the data. Y, H, Y, S and S wrote the manuscript. All authors read and approved the final manuscript.

## Pre-publication history

The pre-publication history for this paper can be accessed here:

http://www.biomedcentral.com/1471-2407/13/258/prepub

## Supplementary Material

Additional file 1: Table S1 List of primer sequences for RT-PCR. Click here for file
